# Correction: Histone variant H2A.Z is needed for efficient transcription-coupled NER and genome integrity in UV challenged yeast cells

**DOI:** 10.1371/journal.pgen.1011646

**Published:** 2025-04-01

**Authors:** Hélène Gaillard, Toni Ciudad, Andrés Aguilera, Ralf E. Wellinger

The funding statement for this article is incorrect. The correct funding statement is as follows: Research and publication of this work was supported by grant PID2022-140466NB-I00 funded by MICIU/AEI/10.13039/501100011033 and FEDER, UE (to HG and RW). Research was additionally supported by grants from the Junta de Andalucía (P20_01220 to RW), the University of Seville (PP2018-10767 and PP2019-13299 to HG), the Spanish Ministry of Science and Innovation (BFU2016-75058-P to AA), the Spanish Ministry of Economy and Competitiveness (BFU2013-42918-P to AA), and the European Regional Development Fund (FEDER). The funders had no role in study design, data collection and analysis, decision to publish, or preparation of the manuscript.
