# Supplementary figures and images for: Histone variant H2A.Z is needed for efficient transcription-coupled NER and genome integrity in UV challenged yeast cells
Source: PLoS Genet. 2024 Sep 10;20(9):e1011300. doi: 10.1371/journal.pgen.1011300 (PMC11414981; doi:10.1371/journal.pgen.1011300)

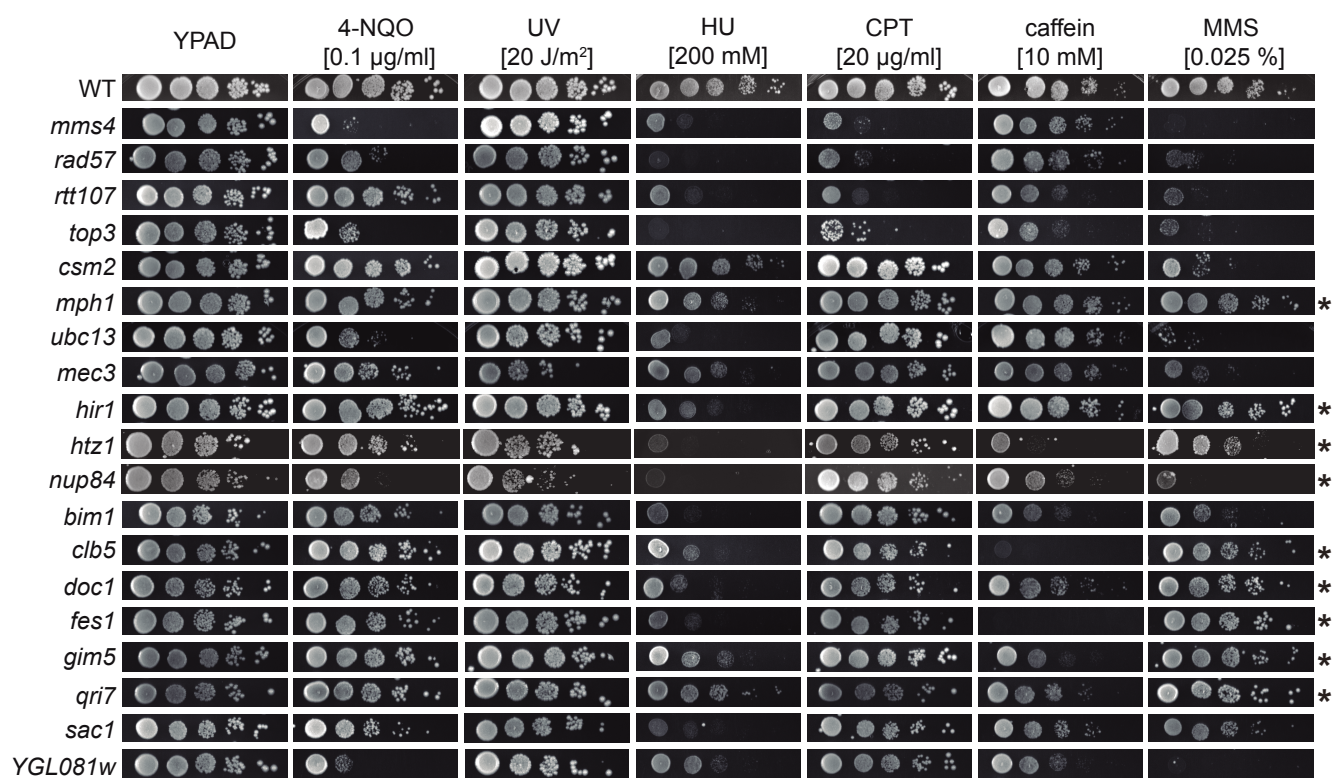

Supporting Figure S1

Supplement: S1 Fig — Growth analysis of wild-type (WT) and 19 selected single mutants upon exposure to different genotoxic agents. Serial dilutions of exponentially growing cultures were spotted on YPAD plate supplemented or not with 0.1 μg/ml 4-nitroquinoline 1-oxide (4-NQO), 200 mM hydroxyurea (HU), 20 μg/ml camptothecin (CPT), 10 mM caffein, or 0,025% methyl methanesulfonate (MMS). Where indicated, plates were irradiated with 20 J/m2 UV-C light. Pictures were taken after incubating the plates in the dark for 3 days. Strains that were selected for subsequent CPD repair analysis are indicated with an asterisk. (PDF) [file pgen.1011300.s001.pdf]

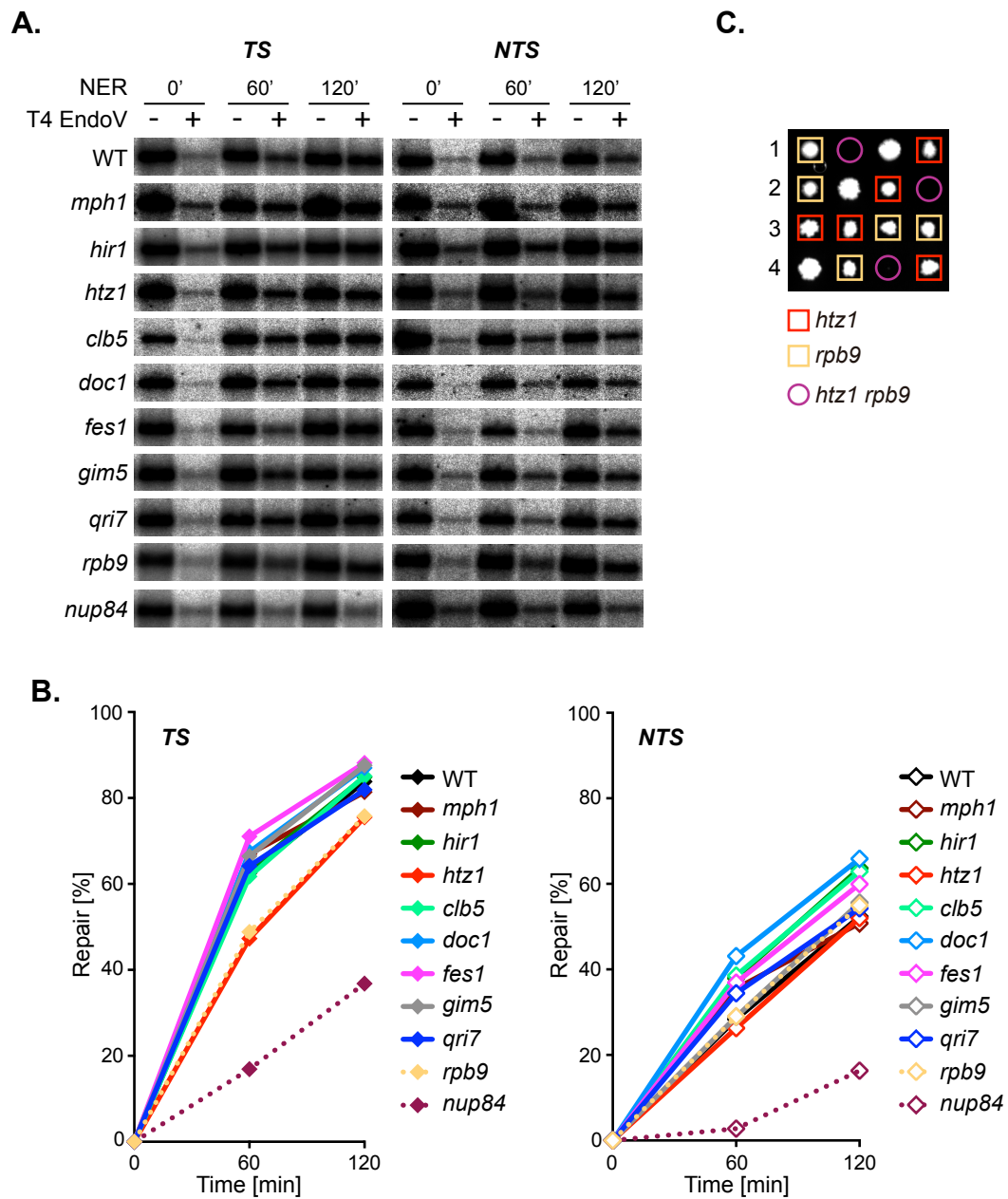

Supporting Figure S2

Supplement: S2 Fig — A. Southern analysis showing the repair of a 4.4-kb (NsiI/PvuI) RPB2 fragment in WT and 8 selected mutants on the transcribed strand (TS) and the non-transcribed strand (NTS). NER-defective nup84Δ and TC-NER defective rpb9Δ strains were used as controls. Non-irradiated DNA and DNA not treated with T4endoV were used as controls. B. Quantification of the CPD repair results shown in (A) is plotted for the TS (left) and NTS (right) (n = 1). C. Tetrad dissection analysis from a htz1Δ rpb9Δ diploid strain. (PDF) [file pgen.1011300.s002.pdf]

**A.**

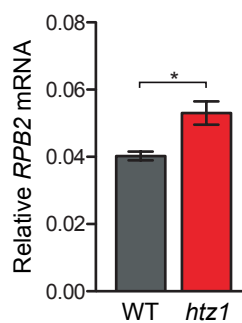

**B.**

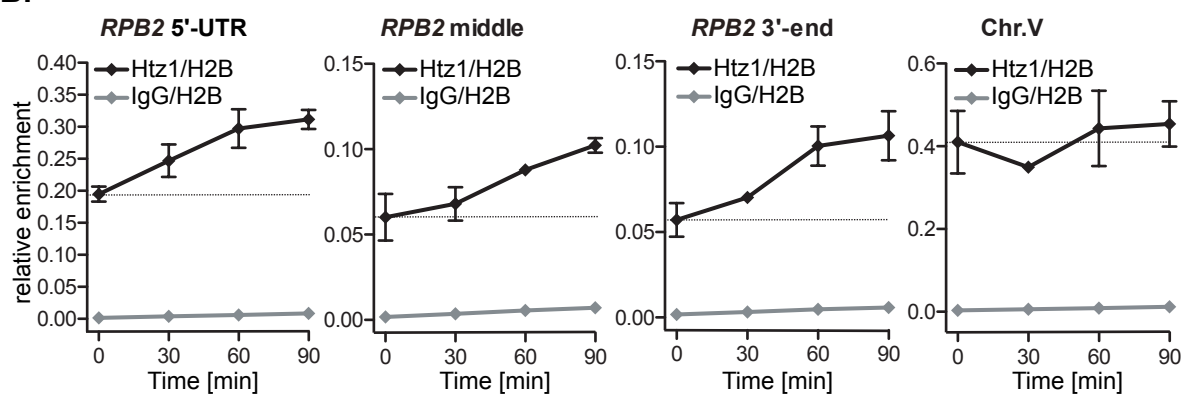

**C.**

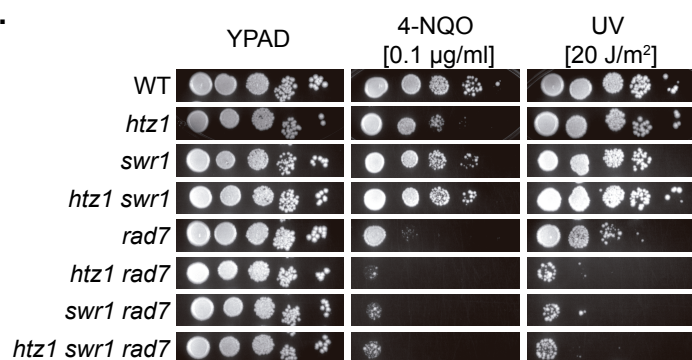

**D.**

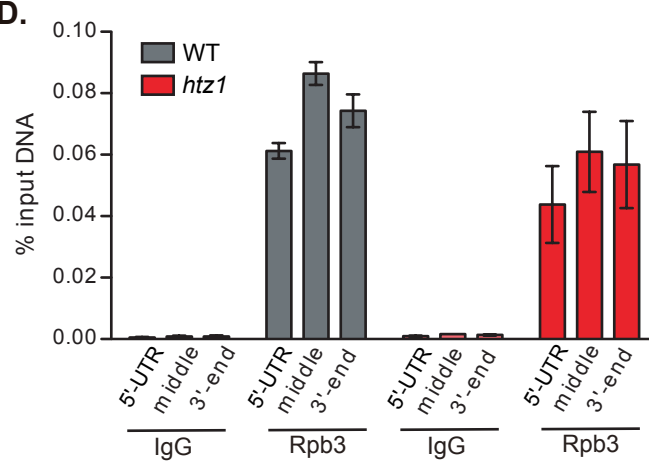

Supporting Figure S3

Supplement: S3 Fig — A. RPB2 expression analysis in WT and htz1Δ as determined by RT-qPCR at the RPB2 middle locus. Average values and SEM are plotted (n = 3). Statistical analyses were performed with a two-tailed unpaired student t test. * P<0.05 B. Analysis of relative H2A.Z enrichment at the 5’-UTR, middle and 3’-end of the RPB2 gene upon UV irradiation. ChIP was performed in a strain bearing tagged versions of Htz1 and H2B. Relative enrichment was obtained by normalization of the immunoprecipitated Htz1 signal with the H2B signal obtained in parallel with the same extract. Non-coding chromosome V region (Chr. V) was used as control. Average values and SEM are plotted for each region (n = 2). Mouse IgG was used as negative control. C. Growth analysis of wild-type (WT) and single, double or triple mutants’ combination of htz1Δ, swr1Δ and rad7Δ upon exposure to 4-nitroquinoline 1-oxide (4-NQO) and UV light. Serial dilutions of exponentially growing cultures were spotted on YPAD plate supplemented or not with 0.1 μg/ml 4-NQO. Where indicated, plates were irradiated with 20 J/m2 UV-C light. Pictures were taken after incubating the plates in the dark for 3 days. D. Percentage of input DNA obtained by ChIP with mouse IgG or Rpb3 antibody in WT and htz1Δ cells at the indicated RPB2 regions. Average values and SEM are plotted for each condition (n = 2). (PDF) [file pgen.1011300.s003.pdf]

**WT conditions**

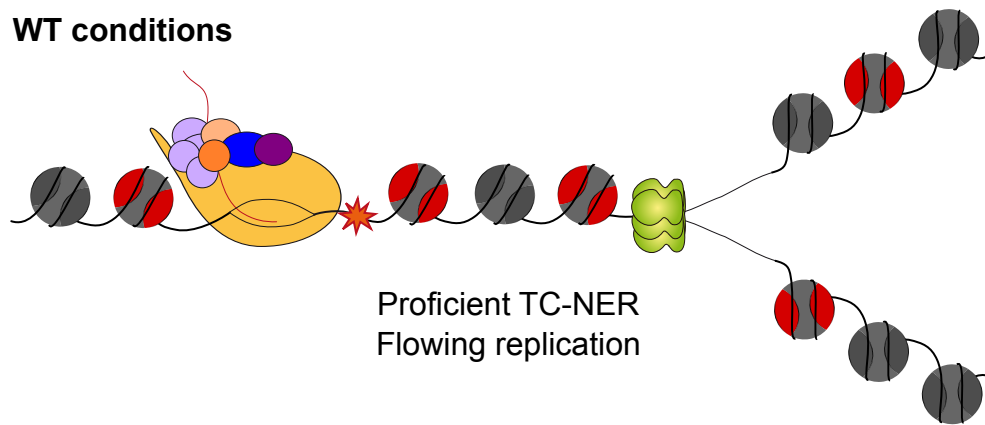

**H2A.Z absence**

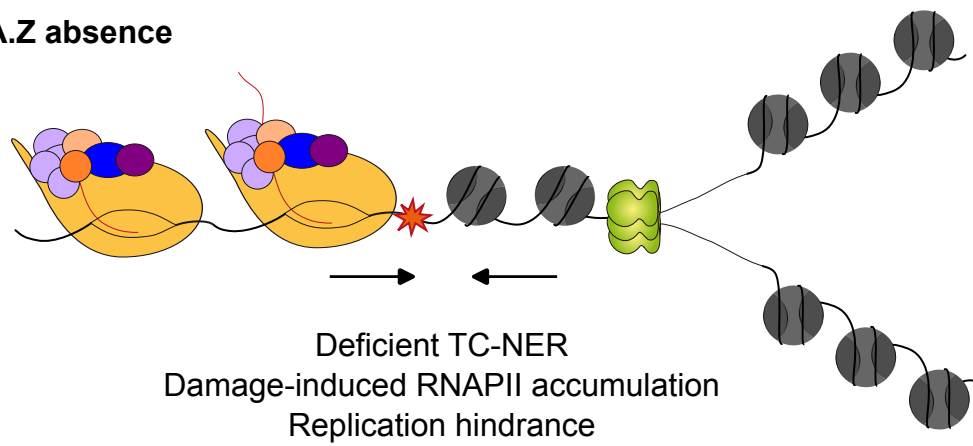

Supporting Figure S4

Supplement: S4 Fig — Schematic drawing illustrating the function of H2A.Z-enriched chromatin in the vicinity of RNAPII stalled at a bulky DNA lesion and at replication forks in wild-type (WT) and htz1Δ cells. The RNAPII is drawn in yellow, the replisome schematized as green helicase and H2A.Z-containing nucleosomes highlighted in red. (PDF) [file pgen.1011300.s004.pdf]
