# Supplementary material for: Histone variant H2A.Z is needed for efficient transcription-coupled NER and genome integrity in UV challenged yeast cells
Source: PLoS Genet. 2024 Sep 10;20(9):e1011300. doi: 10.1371/journal.pgen.1011300 (PMC11414981; doi:10.1371/journal.pgen.1011300)
Supplement: S2 Table — (PDF) [file pgen.1011300.s006.pdf]

Supporting Table S2. Strains used in this study.

Name	Relevant Genotype	Reference
BY4741	*MAT<u>a</u> his3Δ1 leu2Δ0 met15Δ0 ura3Δ0*	Euroscarf
BY4742	*MATα his3Δ1 leu2Δ0 lys2Δ0 ura3Δ0*	Euroscarf
yHG72-1B	*rad7Δ::URA3,* isogenic to BY4741	(1)
yHG72-7D	*rad7Δ::URA3*, isogenic to BY4742	(1)
FYAT269-15D	*MATα-P1LEU2, trp1Δ, leu2Δ, lys2Δ, his3Δ, ura3Δ, arg8Δ*	Alain Nicolas
yHG84-2A	*MATα-P1LEU2*, *rad7Δ::URA3* trp1Δ *met15Δ his3Δ, leu2Δ, ura3Δ*	This study
YBR098W	*mms4Δ::KanMX*, isogenic to BY4741	Euroscarf
YDR004W	*rad57Δ::KanMX*, isogenic to BY4741	Euroscarf
YHR154W	*rtt107Δ::KanMX*, isogenic to BY4741	Euroscarf
YLR234W	*top3Δ::KanMX*, isogenic to BY4741	Euroscarf
YIL132C	*csm2Δ::KanMX*, isogenic to BY4741	Euroscarf
YIR002C	*mph1Δ::KanMX*, isogenic to BY4741	Euroscarf
YDR092W	*ubc13Δ::KanMX*, isogenic to BY4741	Euroscarf
YLR288C	*mec3Δ::KanMX*, isogenic to BY4741	Euroscarf
YBL008W	*hir1Δ::KanMX*, isogenic to BY4741	Euroscarf
YOL012C	*htz1Δ::KanMX*, isogenic to BY4741	Euroscarf
YDL116W	*nup84Δ::KanMX*, isogenic to BY4741	Euroscarf
YER016W	*bim1Δ::KanMX*, isogenic to BY4741	Euroscarf
YPR120C	*clb5Δ::KanMX*, isogenic to BY4741	Euroscarf
YGL240W	*doc1Δ::KanMX*, isogenic to BY4741	Euroscarf
YBR101C	*fes1Δ::KanMX*, isogenic to BY4741	Euroscarf
YML094W	*gim5Δ::KanMX*, isogenic to BY4741	Euroscarf
YDL104C	*qri7Δ::KanMX*, isogenic to BY4741	Euroscarf
YKL212W	*sac1Δ::KanMX*, isogenic to BY4741	Euroscarf
YGL081W	*YGL081wΔ::KanMX*, isogenic to BY4741	Euroscarf
yPM13-10B	*mms4Δ::KanMX rad7Δ::URA3 met15Δ0 LYS2*, isogenic to BY4742	This study
yPM29-1C	*rad57Δ::KanMX rad7Δ::URA3 MET15,* isogenic to BY4742	This study
yPM18-6A	*rtt107Δ::KanMX rad7Δ::URA3*, isogenic to BY4741	This study
yPM31-2C	*top3Δ::KanMX rad7Δ::URA3 lys2Δ0*, isogenic to BY4741	This study
yPM10-2D	*csm2Δ::KanMX rad7Δ::URA3 MET15 lys2Δ0*, isogenic to BY4741	This study
yPM14-10C	*mph1Δ::KanMX rad7Δ::URA3,* isogenic to BY4741	This study
yPM30-2C	*ubc13Δ::KanMX rad7Δ::URA3 MET15 lys2Δ0*, isogenic to BY4741	This study
yPM25-1C	*mec3Δ::KanMX rad7Δ::URA3 met15Δ0 LYS2*, isogenic to BY4742	This study

yPM32-7B	*hir1Δ::KanMX rad7Δ::URA3 MET15*, isogenic to BY4741	This study
yPM33-3B	*htz1Δ::KanMX rad7Δ::URA3 MET15 lys2Δ0,* isogenic to BY4741	This study
yHG187-7B	*htz1Δ::KanMX rad7Δ::URA3 met15Δ0,* isogenic to BY4742	This study
yN84R7-24A	*nup84Δ::KanMX rad7Δ::URA3 MET15*, isogenic to BY4741	(2)
yPM20-15B	*bim1Δ::KanMX rad7Δ::URA3*, isogenic to BY4742	This study
yPM26-8A	*clb5Δ::KanMX rad7Δ::URA3 MET15 lys2Δ0*, isogenic to BY4741	This study
yPM28-4D	*doc1Δ::KanMX rad7Δ::URA3 MET15,* isogenic to BY4741	This study
yPM12-2D	*fes1Δ::KanMX rad7Δ::URA3 MET15 lys2Δ0*, isogenic to BY4741	This study
yPM21-7D	*gim5Δ::KanMX rad7Δ::URA3*, isogenic to BY4742	This study
yPM15-1D	*qri7Δ::KanMX rad7Δ::URA3,* isogenic to BY4741	This study
yPM22-5D	*sac1Δ::KanMX rad7Δ::URA3*, isogenic to BY4741	This study
yPM19-2B	*YGL081wΔ::KanMX rad7Δ::URA3*, isogenic to BY4741	This study
YGL070C	*rpb9Δ::KanMX*, isogenic to BY4741	Euroscarf
yHG127-1	*rad52Δ::NatMX4*, isogenic to BY4741	This study
yHG127-6	*rpb9Δ::KanMX rad52Δ::NatMX4*, isogenic to BY4741	This study
yHG127-4	*htz1Δ::KanMX rad52Δ::NatMX4*, isogenic to BY4741	This study
yHG126-1	*rad18Δ::HIS3MX6*, isogenic to BY4741	This study
yHG126-6	*rpb9Δ::KanMX rad18Δ::HIS3MX6*, isogenic to BY4741	This study
yHG126-8	*htz1Δ::KanMX rad18Δ::HIS3MX6*, isogenic to BY4741	This study
yHG122-1	*rad1Δ::LEU2*, isogenic to BY4741	This study
yHG122-2	*rpb9Δ::KanMX rad1Δ::LEU2*, isogenic to BY4741	This study
yHG122-11	*htz1Δ::KanMX rad1Δ::LEU2*, isogenic to BY4741	This study
yHG223-4	*rad26Δ::HIS3,* isogenic to BY4741	This study
yHG225-3	*htz1Δ::KanMX rad26Δ::HIS3,* isogenic to BY4741	This study
yHG224-3	*rad26Δ::HIS3 rad7Δ::URA3,* isogenic to BY4741	This study
yHG226-4	*htz1Δ::KanMX rad26Δ::HIS3 rad7Δ::URA3,* isogenic to BY4741	This study
YKL160W	*elf1Δ::KanMX,* isogenic to BY4741	Euroscarf
yHG231-9B	*elf1Δ::KanMX,* isogenic to BY4741	This study
yHG231-3C	*htz1Δ::KanMX elf1Δ::KanMX,* isogenic to BY4741	This study
yHG231-8B	*elf1Δ::KanMX rad7Δ::URA3,* isogenic to BY4742	This study
yHG240-3	*htz1Δ::KanMX elf1Δ::KanMX rad7Δ::URA3,* isogenic to BY4741	This study
yHG198-2D	*3MYC-HTZ1 3HA-HTB2*, isogenic to BY4742	This study

YDR334W	*swr1Δ::KanMX*, isogenic to BY4741	Euroscarf
yHG133-5D	*htz1Δ::KanMX LYS2,* isogenic to BY4742	This study
yHG176-6A	*htz1Δ::KanMX swr1Δ::KanMX,* isogenic to BY4741	This study
yHG187-6C	*swr1Δ::KanMX rad7Δ::URA3 met15Δ0,* isogenic to BY4742	This study
yHG187-3C	*htz1Δ::KanMX swr1Δ::KanMX rad7Δ::URA3,* isogenic to BY4741	This study

1. Gaillard H, Tous C, Botet J, González-Aguilera C, Quintero MJ, Viladevall L, et al. Genome-wide analysis of factors affecting transcription elongation and DNA repair: A new role for PAF and Ccr4-not in transcription-coupled repair. PLoS Genet. 2009;5(2):e1000364. PMID: 19197357
2. Gaillard H, Santos-Pereira JM, Aguilera A. The Nup84 complex coordinates the DNA damage response to warrant genome integrity. Nucleic Acids Res. 2019;47(8):4054–67. PMID: 30715474
