# Supplementary material for: Histone variant H2A.Z is needed for efficient transcription-coupled NER and genome integrity in UV challenged yeast cells
Source: PLoS Genet. 2024 Sep 10;20(9):e1011300. doi: 10.1371/journal.pgen.1011300 (PMC11414981; doi:10.1371/journal.pgen.1011300)
Supplement: S3 Table — (PDF) [file pgen.1011300.s007.pdf]

Supporting Table S3. Plasmids used in this study.

Plasmid name	Relevant features	Reference
pRS316-TINV	Ycp pRS316 with *TetO-leu2HOr* and *leu2Δ5'* alleles in inverted orientations	(1)
pARSGLB-OUT	Ycp pFERNU containing the GLB direct-repeat recombination system under the *GAL1* promoter in co-directional orientation with respect to replication.	(2)
pARSGLB-IN	Ycp pFERNU containing the GLB direct-repeat recombination system under the *GAL1* promoter in head-on orientation with respect to replication.	(2)
pWJ1344	Ycp pRS415 expressing a RAD52-YFP fusion protein.	(3)

1. Gonzalez-Barrera S, Garcia-Rubio M, Aguilera A. Transcription and double-strand breaks induce similar mitotic recombination events in *Saccharomyces cerevisiae*. Genetics. 2002;162(2):603–14. PMID: 12399375
2. Prado F, Aguilera A. Impairment of replication fork progression mediates RNA polII transcription-associated recombination. EMBO J. 2005;24(6):1267–76. PMID: 15775982
3. Alvaro D, Sunjevaric I, Reid RJD, Lisby M, Stillman DJ, Rothstein R. Systematic hybrid LOH: a new method to reduce false positives and negatives during screening of yeast gene deletion libraries. Yeast. 2006;23(14–15):1097–106. PMID: 17083134
