# Supplementary material for: Histone variant H2A.Z is needed for efficient transcription-coupled NER and genome integrity in UV challenged yeast cells
Source: PLoS Genet. 2024 Sep 10;20(9):e1011300. doi: 10.1371/journal.pgen.1011300 (PMC11414981; doi:10.1371/journal.pgen.1011300)
Supplement: S4 Table — (PDF) [file pgen.1011300.s008.pdf]

Supporting Table S4. Primers used in this study.

Name	Sequence	Usage
RAD1 A	GACGTTGGTAGAGCATTTGC	Amplification *rad1Δ::LEU2* fragment
RAD1 D	ATGGAATCCCTGCTGCCCA	Amplification *rad1Δ::LEU2* fragment
RAD26 A	TGATTATTTATGAAAATTGGCCTGT	Amplification *rad26Δ::HIS3* fragment
RAD26 D	AACGGGTATCTCTACTATCCCATTC	Amplification *rad26Δ::HIS3* fragment
RAD7 up	ATCAATGTCAATGTTCCCTCTTC	Amplification *rad7Δ::URA3* fragment
RAD7 down	ATCTAAACCGTTTAACGTGAGAG	Amplification *rad7Δ::URA3* fragment
Rpb2A	TCTTGGAATAATAACTTCGCGGC	*RPB2* probe
Rpb2B	GGTGGATGACAAGATACATGCC	*RPB2* probe
RPB2 5'-UTR up	AACACGACAGCGGAATAAGACA	Real-time qPCR
RPB2 5'-UTR down	GTTTGTGTTCTTTTTCTGGTCCTAATT	Real-time qPCR
RPB2 middle up	CCACTGGAAGATTACGTACCACATC	Real-time qPCR
RPB2 middle down	GCCAAACACCATTGACGAAGA	Real-time qPCR
RPB2 3'-end up	CAACGTACAAACACCTTAAGAATGAAAC	Real-time qPCR
RPB2 3'-end down	CAGGCGCAATTAGACCATCA	Real-time qPCR
intergenic V1	TGTTCCTTTAAGAGGTGATGGTGAT	Real-time qPCR
intergenic V2	GTGCGCAGTACTTGTGAAAAC	Real-time qPCR
SCR1_426 FW	GATCGCTTCGGCGGTTTAA	Real-time qPCR
SCR1_483 REV	GGCCACAATGTGCGAGTAAAT	Real-time qPCR
