# Supplementary material for: Histone variant H2A.Z is needed for efficient transcription-coupled NER and genome integrity in UV challenged yeast cells
Source: PLoS Genet. 2024 Sep 10;20(9):e1011300. doi: 10.1371/journal.pgen.1011300 (PMC11414981; doi:10.1371/journal.pgen.1011300)
Supplement: Attachment — Submitted filename: Rebuttal_Gaillard.docx [file pgen.1011300.s010.docx]

Comments to the Authors:

Reviewer #1: The manuscript by Gaillard et al. reports the identification of the histone variant H2A.Z as a component in the resistance of yeast cells to 4-NQO and UV. The authors further demonstrate that H2A.Z plays a significant role in repairing UV lesions in the transcribed strand and, to a lesser extent, in the non-transcribed strand of the RPB2 gene, indicating a role for H2A.Z in TC-NER and GG-NER. The elimination of H2A.Z is epistatic to the deletion of Swr1, consistent with a previous study (doi: 10.1093/nar/gkt688) showing that Swr1 facilitates NER by recruiting H2A.Z to chromatin. These results support the idea that Swr1, along with H2A.Z, facilitates TC-NER and GG-NER by affecting their common substrates—lesions in chromatin. In line with its role in NER, H2A.Z appears to constrain recombination associated with UV damage, especially in situations where the transcription machinery collides with the DNA replication machinery.

The research was well planned and conducted, and the conclusions are largely well-supported by the experimental data. However, the findings presented are not novel. As noted by the authors, H2A.Z has been well-known to be implicated in numerous processes, including its role in NER. Therefore, I do not believe the findings are significant enough for publication in PLoS Genetics.

The study by R. Waters and colleagues to which the referee is referring nicely shows that the presence of H2A.Z nucleosomes in a repressed promoter prior to UV irradiation facilitates GG-NER by recruiting Gcn5 and promoting H3 acetylation and Rad14 binding (Yu et al, NAR, 2013). Notably, this study shows that H2A.Z does not influence GG-NER at a repressed region that is not enriched in H2A.Z nucleosomes, and does not assess the repair at transcribed genes. Therefore, it does not provide data to conclude that H2A.Z plays a role in NER as suggested by the referee. Our finding that H2A.Z is required for TC-NER, and favours GG-NER on the NTS of an active gene (not enriched in H2A.Z nucleosomes), is novel and has, to the best of our knowledge, never been shown before.

We have revised the mention and the discussion of the study by Yu and colleagues in our revised manuscript. During the revision of this part of the manuscript, we found a mistake (line 127), which has been corrected. In addition, we now also discuss a posterior work demonstrating that the GG-NER factor Rad7-Rad16 initiates GG-NER by recruiting Gcn5 and promoting H3 acetylation in non-transcribed regions and in the NTS of active genes (Yu et al, Genome Research, 2016). We acknowledge the referee's comments, which helped us improve the discussion of our data.

Reviewer #2: The manuscript by Gaillard et al screens >5K individual mutants to identify and characterize the involvement, and requirement of the gene encoding a highly conserved H2A.Z variant histone for efficient transcription-coupled NER. This is confirmed by well-designed plating assays and strand-specific DNA repair assays, and genomic instability assayed with reporter constructs. This is a well laid out study overall that ascribes a role of this histone variant selectively in TC-NER. The findings should be of interest fairly broadly. That said, there are some issues that should be addressed before the manuscript is ready.

Moderate issues.

Lines 110-113. What is the rationale or use in classifying mutants by categories? This seems arbitrary, as written,

We completely agree with the referee. The classification in categories is pointless and we have eliminated this classification in our revised manuscript. Many thanks for helping us improve our article.

Do authors see or expect to see changes in mRNA production of the target genes in the absence of H2A.Z? Could at least some defects in TC-NER be explained by changes in transcription?

Because TC-NER is strictly dependent on active transcription, the referee is right and transcriptional defects could lead to deficient repair. Previous genome-wide expression analyses of htz1∆ yeast cells by microarray did not show differential expression of the RPB2 gene (Morillo-Huesca et al, PLoS One, 2010). To assess RPB2 mRNA levels in our experimental conditions, we have now performed RT-qPCR analyses in WT and htz1∆ cells. The results, which are included as panel A in our revised Supporting Fig. S3, indicate a small increase in expression level in htz1∆ as compared to WT. These results are appropriately described and discussed in our revised manuscript.

Lines 137-141. An alternative explanation is that GG-NER can be also involved in the same repair in addition to TC-NER components. This should be considered when making conclusions from the data. This could potentially explain lack of complete epistasis as shown in the next section, for example.

Lines 137-141 of the originally submitted manuscript describe the epistasis analysis of htz1 with a HR factor (rad52), post-replicative repair factor (rad18), and NER endonuclease (rad1). The Rad1 endonuclease is required for NER (independently of the sub-pathway) and participates in other repair processes, such as the HR sub-pathway of single strand annealing, for example. If we understand correctly the query of the referee, the use of 'NER endonuclease' was confusing, so we have removed 'NER' and now mention that it is required for both TC-NER and GG-NER. About the following section (sensitivity of htz1 rad7 mutants to different genotoxic agents), we have rephrased the conclusion of our data in the results.

I was not able to find information on, in authors’ opinion, how H2A.Z specifically contributes to TC-NER. Is this by recruiting some partners, changing nucleosome biophysical properties, enforcing Pol II termination, etc. What authors think happens should be specified in Discussion more clearly. For example, Line 268 states the fact, but offers no discussion as to why.

We have revised the discussion and the possible ways in which H2A.Z could contribute to TC-NER and favors GG-NER of the NTS have ben indicated. However, we prefer not to develop this further to avoid speculation. We are grateful for this comment, which helped us to improve the discussion of our manuscript.

Perhaps the more substantial data presentation issue has to do with ChIP experiments, which are currently shown as either arbitrary units or ratios between antibodies, neither of which is informative. ChIP data should be presented as percent of input for each individual antibody, with ratios presented optionally. A no-antibody control should also be included. This applies to figure S3A and main 2C.

The ChIP experiments of our study aim at detecting small changes in RNAPII and Htz1 occupancy in response to UV-induced damage. Because of the inherent variability across samples (number of cells, efficiency of cell breakage, sonication efficiency, recovery of chromatin extract), we have used a non-coding region of chromosome V as internal control in these time-course experiments. This region, which is well established as negative control in S. cerevisiae, is commonly used to express the relative occupancy RNAPII or other transcription factors at transcribed genes (see for example Grzechnik et al, Genes & Dev., 2015; Qiu et al, Genome Research, 2016; Sen et al, Mol Cell Biol, 2019). To display our data in a more informative way, we have now used the comparative Ct method (2^-∆∆Ct^ method) to calculate the relative Rpb3 enrichment as compared to the chromosome V non-coding region (revised Fig. 2C). In addition, we have included ChIP results obtained using mouse IgG instead of the Rpb3 antibody, which are plotted as % of input DNA in the new panel D of Supporting Fig. S3.

We agree with the referee that, due to the different binding affinity of antibodies, the ratio between IP obtained with different antibodies is not informative by itself (at one single locus or time-point), but it is important to acknowledge that the changes in this ratio between loci or conditions are informative. Indeed, ChIP analyses of histone variants or histone modifications are commonly normalized with the signal obtained from unmodified canonical histone in the same extract (examples can be found in Li et al, PNAS, 2005; Creyghton et al, Cell 2008; Millan-Zambrano et al, Mol Cell, 2018; Santos-Rosa et al, Mol Cell, 2021). Therefore, the changes in the ratio of Htz1 IP to H2B IP signals obtained in parallel from the same chromatin sample are meaningful. The revised Supporting Fig. S3B displays the relative enrichment of Htz1 over the H2B IP signal for each region, without normalization to the non-irradiated sample. The enrichment of IgG IP over H2B IP is now included as control.

The results, M&M and figure legends sections have been appropriately revised and raw ChIP data are now provided as supporting information. We are thankful for this comment, which allowed us to correct a mistake (5 min time-point at the 5'-UTR of WT) and to improve our manuscript.

Minor points.

Are transcription-coupled NER and genome integrity roles of H2A.Z coming from the same mechanism or are two independent roles? The title seems to suggest the latter, but it is not clear whether this distinction is made or really should be made based on the presented data.

As exposed in the discussion, which has been revised, we propose that H2A.Z contributes to restrain genome instability by promoting efficient TC-NER and avoiding the accumulation of stalled RNAPII. The title has also been revised and now reads: 'Histone variant H2A.Z is needed for efficient transcription-coupled NER and genome integrity in UV challenged yeast cells'.

Is it necessary to spend a good portion of Introduction on Elf1, which is barely mentioned in Results?

The introduction of Elf1 has been reduced as suggested. Thank you for improving the flow of our manuscript.

Fig 1A is described as showing a portion of 820 mutants with growth impairment when combined with rad7Delta. This is not seen in the actual Fig 1A example.

In the plate that is shown as example in Fig. 1A, the strain highlighted with an orange circle showed synthetic 4-NQO sensitivity when combined with the rad7∆ deletion. This is now clearly described in the figure legend.

Fig 2A. It appears that rad7 individual mutant grows better than WT in the presence of MMS and Mnd. Do authors have an explanation and/or a more representative picture if it is an artifact?

Many thanks for this comment, more representative pictures are now provided in Fig. 2A.

Fig S1. Are these single mutants? Might be useful to highlight this in the legend. Also, nup84 mutant mentioned in the main text should probably be highlighted on the figure.

This is correct, Supporting Fig. S1 shows sensitivity of single mutants to different genotoxic agents. This is now stated in the legend and in the main text. The nup84∆ mutant is now highlighted in the figure. Many thanks for pointing out these omissions.

Line 44. What data suggest “chromatin plasticity” conclusion and what this means?

We intent to refer to the chromatin changes which are required for efficient repair and to prevent DNA damage induced genome instability. We have rephrased this sentence to clarify its meaning. The sentence now reads: 'Our findings about the contribution of histone variant H2A.Z to the repair of UV damage further highlight the importance of chromatin environment for the maintenance of genome integrity.'

Line 51. Delete “severely” or delete the entire part of the sentence starting with “and thus”.

This part of the sentence has been removed as suggested. Thank you.

Line 73. Delete “trigger”.

Deleted as suggested.

Line 203. Delete “Interestingly”. The same can be done in most other instances throughout.

The adverb has been deleted at lines 203 and at two other instances.

Line 227. Delete “apparently”.

Deleted as suggested.

Line 262. “Unstabilize”?

Corrected and now reads ''destabilize''.

Some language should be proofread throughout, for example:

Line 50. “DNA damages generate” -> “DNA damage generates”

Corrected.

Lines 69-71. “In GG-NER” should be moved to after “while” to read: “while in GG-NER, a specialized DNA damage recognition complex…”

Corrected.

Line 104. “baring” should be “bearing”

Corrected. This mistake has been corrected throughout the manuscript.

Line 109. “in” should be “into”

This sentence has been deleted by removing the categories.

Line 117. “encode for” should be either “encode” or “code for”

Has been changed to ''encode''.

Line 181. “liked” should be “linked”

Corrected.

Line 191. “positioned”

Corrected.

Line 209. Delete “a”

Corrected.

Line 216. Delete “thus”.

Corrected.

Line 220. “raised” should be “rose”

Corrected.

Line 221. “Substantial”

Corrected.

Line 222. Delete “do”.

Corrected.

Line 258. Should be “DNA damage sites”

Corrected.

Line 259. “region” should be “regions”.

Corrected.

Line 260. “in” should be “into”.

Corrected.

Line 269. “eventhough” should be “even though”.

Corrected. Many thanks for proofreading our manuscript.

Reviewer #3: The manuscript by Gaillard et al describes a very nice initial characterization of a novel role for the H2A.Z histone variant in TC-NER. The authors conducted a systematic screen for increased 4NQO sensitivity in rad7 background, and found a number of interesting hits. They focused the subsequential analyses on arguably the most interesting of them all, HTZ1 encoding the H2A.Z homolog in S. cerevisiae. This particular histone variant has been shown previously to play multiple roles in genome stability, but never before in TC-NER. The work reported in this manuscript is going to be impactful because it uncovered yet another important facet of HA2.Z’s function in genome protection, so for this reason, I believe this work would receive a good amount of attention from a broad audience. The results of the screen itself (several additional genes) are also quite valuable as a resource that will be appreciated by the DNA repair community. The text is very nicely written and the data presented are quite clear and straightforward. I do not have any concerns about this work, and I only offer very minor suggestions for revisions to the text listed below.

Line 22: roadblocks (plural)

Corrected. Many thanks for the proofreading.

Line 55: structurally

Corrected.

Line 74: in (lower case i)

Corrected.

Line 104: bearing (not baring). “baring” is also incorrectly used as at other parts of the manuscript.

Corrected throughout the manuscript.

Line 155: It looks to me in Fig. 2B like the effect on the NTS is also pretty substantial. How would this look like in a GG-NER defective mutant? Perhaps add this point somewhere in the Discussion?

Previous reports have shown that NTS repair is severely compromised in GG-NER defective mutants as compared to wild-type cells (Verhage et al, Mol Cell Biol, 1996; Gaillard et al, PLoS Genetics, 2009, Yu et al, Genome Research, 2015). Although the NTS repair defects observed in htz1∆ are quite moderate as compared to wild-type cells, we agree with the referee that they deserve to be discussed. We have now revised how this data is described and discussed in our manuscript. Thanks for helping us to improve our article.

Line 176: “these results indicate that H2A.Z is required for proficient TC-NER”. I am not so sure about the use of “required” in this sentence. This is a subtle point, but I think this effect is better described as "contributes to" TC-NER, avoiding the term "required", which is potentially confusing to some readers. Consider changing to: “these results indicate that H2A.Z is an important contributor to proficient TC-NER”.

Changed as suggested. In the same line, we have modified the title and abstract accordingly.

Line 181: “have liked the absence of H2A.Z with increased genome instability”; replace with have liked the absence of H2A.Z to increased genome instability”

Changed as suggested.

Line 221: Substantial

Corrected.

Line 262: destabilize

Corrected.

Line 275: Swr1 (protein notation)

Corrected. Thanks again for your help.

Have all data underlying the figures and results presented in the manuscript been provided?
Large-scale datasets should be made available via a public repository as described in the PLOS Genetics [data availability policy](http://journals.plos.org/plosgenetics/s/data-availability), and numerical data that underlies graphs or summary statistics should be provided in spreadsheet form as supporting information.

Reviewer #1: Yes

Reviewer #2: No: Not clear if ChIP-qPCR raw data were provided

ChIP-qPCR raw data is now provided in spreadsheet form as supporting information, together with the raw data of repair, recombination and Rad52-foci experiments.

Reviewer #3: Yes
